# A Prospective Randomised Comparative Study of the Effect of Propofol and Sevoflurane on Liver and Renal Function Tests in Patients Undergoing Spine Surgeries

**DOI:** 10.7759/cureus.110220

**Published:** 2026-06-04

**Authors:** Sumita Kumari, Venkatesha J, Ram Badan Singh, Anurag Sahu

**Affiliations:** 1 Department of Anaesthesiology, Institute of Medical Sciences, Banaras Hindu University, Varanasi, IND; 2 Department of Neurosurgery, Institute of Medical Sciences, Banaras Hindu University, Varanasi, IND

**Keywords:** anaesthesia, hepatorenal function, postoperative nausea and vomiting, propofol, sevoflurane

## Abstract

Background and aim

Thoracolumbar spine surgeries involve physiological stress that may influence hepatic and renal parameters. This study aimed to compare the effects of propofol-based anaesthesia with sevoflurane-based anaesthesia on liver function tests (LFTs), renal function tests (RFTs) and early postoperative recovery parameters in patients undergoing elective thoracolumbar spine surgery.

Materials and methods

This prospective randomised comparative study included 56 adult patients classified as American Society of Anesthesiologists (ASA) physical status I-II, who were randomly allocated into two groups (n = 28 each). Group P received intravenous (IV) propofol infusion (100-200 µg/kg/minute), while Group S received inhalational sevoflurane (0.8-1.5 minimum alveolar concentration {MAC}). Primary outcomes included serum aspartate aminotransferase (AST), alanine aminotransferase (ALT) and creatinine, measured preoperatively, immediately after extubation and at 24 hours postoperatively. Secondary outcomes included intraoperative hemodynamic stability, Ramsay Sedation Scale (RSS) scores and the incidence of postoperative nausea and vomiting (PONV).

Results

Both groups maintained stable intraoperative hemodynamics, with no significant differences (p > 0.05). At 24 hours, Group S demonstrated significantly higher AST, ALT and creatinine levels compared to Group P (p < 0.001). The incidence of PONV was also significantly higher in Group S (66.7% versus 33.3%; p = 0.029), while sedation scores remained comparable.

Conclusion

Propofol-based anaesthesia was associated with better early postoperative hepatorenal preservation and a lower incidence of PONV compared to sevoflurane, with similar intraoperative hemodynamic stability.

## Introduction

Spine surgeries for degenerative, traumatic and neoplastic conditions are often long and technically demanding procedures associated with significant physiological stress and extensive tissue manipulation. Such procedures involve prone positioning, substantial blood loss and prolonged exposure to anaesthesia, which can affect hemodynamic stability and end-organ perfusion at the systemic level. Maintaining intraoperative hemodynamic stability and preserving the functional integrity of vital organs are the primary goals of anaesthetic management in such cases. Among the organs that may be affected, the liver and kidneys are of particular importance because of their roles in drug metabolism, detoxification and excretion [1,2]. These organs are highly sensitive to surgical stress, alterations in perfusion dynamics and inflammatory responses, which may lead to transient or clinically significant dysfunction [1,3].

An anaesthetic maintenance agent is a key determinant of perioperative organ protection. Commonly used agents in contemporary anaesthesia include propofol and sevoflurane for the maintenance of general anaesthesia. Propofol is an intravenous (IV) drug with a rapid onset and short context-sensitive half-time, and its favourable recovery profile and inherent antiemetic effect make it a preferred agent for total intravenous anaesthesia (TIVA) [4]. Propofol has been hypothesised to exert organ-protective effects through antioxidant, anti-inflammatory and hepatic perfusion-related mechanisms; however, these mechanisms remain incompletely established and may vary according to surgical context, patient physiology and perioperative hemodynamic conditions [5]. In contrast, sevoflurane, an inhalational volatile anaesthetic, is widely used due to its ease of titration, rapid induction and emergence and minimal airway irritation [5]. However, concerns remain regarding its metabolism and potential effects on hepatic enzymes and renal function, particularly with prolonged exposure [6].

Spine surgeries may further increase the risk of organ dysfunction. Prone positioning, commonly used in thoracolumbar procedures, can elevate intra-abdominal and intrathoracic pressures, leading to reduced venous return, decreased cardiac output and compromised renal and hepatic perfusion. These changes may predispose patients to subclinical organ injury, reflected by alterations in biochemical markers such as liver transaminases and serum creatinine [7]. Additional contributing factors include fluid shifts, blood loss and neurohormonal responses to surgical stress, highlighting the importance of selecting anaesthetic techniques with minimal adverse physiological impact [8].

In addition to organ function, postoperative recovery characteristics are important determinants of overall outcomes. Postoperative nausea and vomiting (PONV) is a common and distressing complication that may delay recovery and prolong hospital stay. Propofol is known to have antiemetic properties, whereas volatile agents such as sevoflurane are associated with a higher incidence of PONV. The depth and quality of postoperative sedation also influence early neurological assessment, which is particularly important in spine surgery for the timely evaluation of motor and sensory function. Therefore, the choice of anaesthetic maintenance should consider both intraoperative stability and postoperative recovery quality [9].

Although both propofol and sevoflurane are considered safe and effective, there remains ongoing debate regarding their comparative effects on hepatorenal function and postoperative recovery outcomes. Existing literature presents conflicting findings, with some studies demonstrating similar safety profiles and others suggesting advantages of one agent over the other depending on the surgical context and patient characteristics [10,11]. This lack of consensus highlights the need for further comparative evaluation, particularly in high-risk surgical settings such as spine surgery, where physiological stress and organ preservation are critical considerations [12,13].

In this context, the present prospective randomised comparative study aimed to assess and compare the effects of propofol- and sevoflurane-based anaesthesia on liver function tests (LFTs) and renal function tests (RFTs) in patients undergoing elective thoracolumbar spine surgery. In addition, intraoperative hemodynamic stability and early postoperative recovery parameters, including sedation and PONV, were evaluated to provide a comprehensive assessment of both anaesthetic techniques. The findings are intended to inform evidence-based anaesthetic selection and optimise perioperative management strategies in this high-risk surgical population [14,15].

Objectives of the study

The primary objective of this study was to compare the effects of propofol-based anaesthesia with sevoflurane-based anaesthesia on early postoperative hepatorenal function in adult patients undergoing elective thoracolumbar spine surgery, as assessed by serum aspartate aminotransferase (AST), alanine aminotransferase (ALT) and creatinine levels measured preoperatively, immediately after extubation and 24 hours postoperatively.

The secondary objectives were to compare intraoperative hemodynamic stability between the two anaesthetic techniques, assessed using heart rate (HR) and mean arterial pressure (MAP), and to evaluate early postoperative recovery outcomes, including Ramsay Sedation Scale (RSS) scores, the incidence of postoperative nausea and vomiting, rescue antiemetic requirement and time to first rescue antiemetic.

## Materials and methods

Study design and ethical approval

This prospective randomised comparative study was conducted after the approval of the Institutional Ethics Committee of the Institute of Medical Sciences, Banaras Hindu University (BHU) (approval number: IMS/IEC/2024/2532), approved on 09/10/2024. Written informed consent was obtained from all participants before inclusion. The study was conducted in accordance with the ethical principles of the Declaration of Helsinki and reported according to the Consolidated Standards of Reporting Trials (CONSORT) guidelines.

Study population

A total of 56 adult patients aged between 18 and 65 years, classified as American Society of Anesthesiologists (ASA) physical status I or II, from October 2024 to January 2026, were included. All patients were scheduled for elective thoracolumbar spine surgery in the Institute of Medical Sciences, Banaras Hindu University (BHU), Varanasi, which is a tertiary care teaching hospital. Only patients with baseline aspartate aminotransferase (AST) (5-40 IU/L), alanine aminotransferase (ALT) (5-40 IU/L) and serum creatinine (0.6-1.2 mg/dL) within normal reference ranges were included. Patients with pre-existing hepatic or renal disease, those receiving hepatotoxic or nephrotoxic drugs and those undergoing surgeries for more than 120 minutes were excluded to minimise confounding variables.

Sample size and randomisation

Sixty patients were assessed for eligibility, of whom four were excluded. The remaining 56 patients were randomly allocated into two equal groups (n = 28 each) using a computer-generated random number sequence with block randomisation. Allocation concealment was ensured using sequentially numbered, sealed opaque envelopes, which were opened at the time of induction.

Sample size was calculated using the formula for the comparison of two independent means, based on postoperative aspartate aminotransferase (AST) levels at 24 hours as the primary outcome variable. The calculation was based on data reported by Sahin et al., with mean AST values of 34.5 ± 3.19 IU/L in the propofol group and 45.57 ± 4.89 IU/L in the sevoflurane group [11]. The following formula was used: n = 2σ²(Z₁₋α/₂ + Z₁₋β)² / d², where σ represents the pooled standard deviation and d represents the expected mean difference between groups. A two-sided significance level of 0.05 and a power of 90% were used, corresponding to Z₁₋α/₂ = 1.96 and Z₁₋β = 1.282. Based on an expected mean difference of 11.07 IU/L and a pooled standard deviation of 4.13 IU/L, the required sample size was 26 patients per group. To compensate for an anticipated 5% loss to follow-up, this was increased to 28 patients per group, resulting in a final total sample size of 56 participants. The blinding of the anaesthesiologist was not feasible due to the nature of the interventions. Therefore, the study was conducted as an assessor-blinded trial, where patients, postoperative outcome assessors and laboratory personnel were blinded to group allocation.

Study groups

The participants were allocated into two groups. Group P received a maintenance of anaesthesia with intravenous propofol infusion at 100-200 µg/kg/minute, while Group S received inhalational sevoflurane at 0.8-1.5 minimum alveolar concentration (MAC). The doses were titrated based on hemodynamic responses, including heart rate (HR) and mean arterial pressure (MAP), to maintain an adequate depth of anaesthesia. The depth of anaesthesia was assessed clinically using intraoperative hemodynamic parameters, including heart rate and mean arterial pressure. Bispectral index (BIS) or other processed electroencephalographic monitoring was not used in this study. Propofol and sevoflurane doses were titrated within the predefined study ranges according to hemodynamic responses and institutional anaesthetic practice. Surgical positioning, intraoperative monitoring, anaesthetic maintenance targets and postoperative analgesic protocols were standardised across both study groups to minimise procedural variability.

Perioperative surgical variables

The duration of surgery, estimated intraoperative blood loss, intraoperative fluid administration and the duration of anaesthesia were recorded for all patients to ensure perioperative comparability between groups. Baseline patient characteristics, including age, sex, ASA physical status and preoperative biochemical parameters, were assessed before randomisation. Standardised institutional protocols were followed for patient positioning, intraoperative monitoring, fluid management, anaesthetic induction, maintenance and postoperative analgesia to minimise intergroup variability.

Anaesthetic protocol

All patients received standardised intravenous premedication with midazolam (30-70 µg/kg) and fentanyl (1-2 µg/kg). Intraoperative analgesia was maintained with intermittent doses of fentanyl as required; postoperative analgesia was maintained by IV paracetamol 1 g eight hourly. The induction of anaesthesia was performed using propofol (2 mg/kg) and vecuronium (0.1 mg/kg) to facilitate endotracheal intubation.

Patients were positioned with appropriate precautions for prone positioning. Perioperative fluid management was standardised using isotonic crystalloid solutions. Perioperative fluid was managed using isotonic crystalloid solutions administered at the rate of 1.5-2.5 mL/kg/hour and regulated according to patient requirements, estimated blood loss and hemodynamic parameters. Neuromuscular blockade was reversed using neostigmine (0.05 mg/kg) and glycopyrrolate (0.01 mg/kg), and extubation was performed after adequate recovery of spontaneous respiration and protective airway reflexes.

Intraoperative monitoring

Continuous intraoperative monitoring included heart rate (HR), mean arterial pressure (MAP), peripheral oxygen saturation (SpO₂), end-tidal carbon dioxide (EtCO₂), temperature, urine output monitoring and electrocardiography (ECG). Measurements were recorded at baseline and at 15-minute intervals throughout the intraoperative period.

Outcome measures

Primary outcomes included liver function tests, namely, aspartate aminotransferase (AST) and alanine aminotransferase (ALT), and renal function assessed by serum creatinine. These parameters were measured preoperatively, immediately after extubation and at 24 hours postoperatively.

Secondary outcomes included intraoperative hemodynamic stability and postoperative nausea and vomiting (PONV), which was assessed using a four-point ordinal scale (0 = no symptoms, 1 = nausea, 2 = retching and 3 = vomiting) within the first 24 hours postoperatively. The requirement for rescue antiemetic and time to first rescue antiemetic were also recorded. Rescue antiemetic therapy was administered in patients who developed retching or vomiting or persistent nausea requiring pharmacological treatment, according to institutional postoperative care protocols. Sedation was assessed at 30 minutes post-extubation using the Ramsay Sedation Scale score.

Follow-up and data collection

All patients were followed for 24 hours postoperatively. Biochemical and clinical parameters were recorded at predefined intervals.

Statistical analysis

Statistical analysis was performed using IBM SPSS Statistics for Windows version 25.0 (IBM Corp., Armonk, NY). Data normality was assessed using the Shapiro-Wilk test. Continuous variables were expressed as mean ± standard deviation and compared between groups using the independent samples Student’s t-test when assumptions for parametric testing were satisfied. Before parametric analysis, normality was assessed using the Shapiro-Wilk test, and the homogeneity of variance was assessed using Levene’s test. When Levene’s test indicated equal variances (p > 0.05), equal variances were assumed; when the homogeneity of variance was violated (p < 0.05), the ‘equal variances not assumed’ result was used. Non-normally distributed variables were analysed using the Mann-Whitney U test, as appropriate. Intragroup comparisons over time were performed using repeated measures analysis of variance (ANOVA). Categorical variables were analysed using the chi-square test or Fisher’s exact test, as appropriate, and were presented as frequencies and percentages. To address within-patient biochemical changes, additional patient-wise change-from-baseline analyses were performed. For each patient, changes in AST, ALT and serum creatinine were calculated from preoperative baseline to post-extubation and from preoperative baseline to 24 hours postoperatively. The magnitude of change was compared between the propofol and sevoflurane groups using Welch’s t-test. These analyses were performed to avoid reliance solely on pooled absolute postoperative values and to identify whether postoperative biochemical differences reflected patient-level changes from baseline. A p-value of <0.05 was considered statistically significant, and 95% confidence intervals were calculated where applicable.

## Results

Demographic and baseline characteristics

A total of 56 patients were included in the study, with 28 patients in Group P (propofol) and 28 patients in Group S (sevoflurane). All patients completed the study and were included in the final analysis.

**Figure 1:**
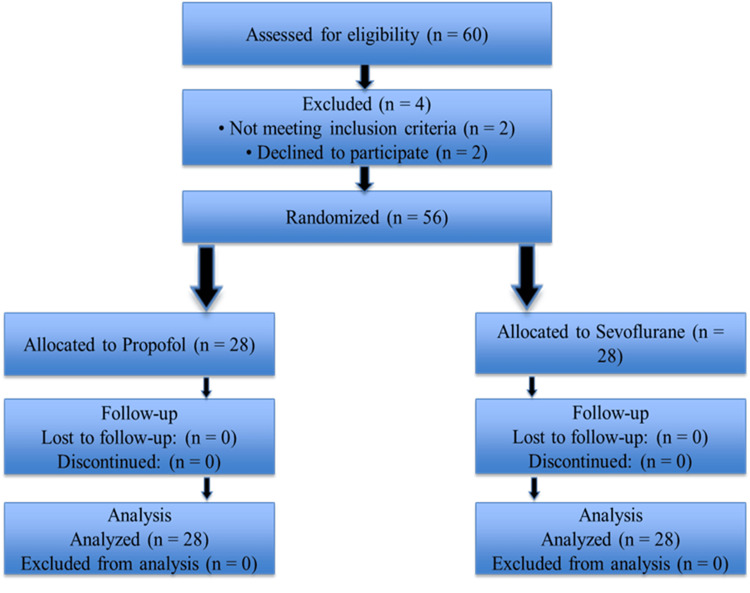
CONSORT Flow Diagram of Patient Enrollment, Allocation, Follow-Up and Analysis CONSORT: Consolidated Standards of Reporting Trials

Baseline demographic characteristics showed no statistically significant differences between groups. Age distribution did not differ significantly (p = 0.783). Sex distribution was comparable (χ² = 0.074, degree of freedom {df} = 1 and p = 0.786), with men comprising 58.9% of the study population. ASA physical status distribution was also similar (χ² = 0.074, df = 1 and p = 0.786). The flow of the participants is presented in Figure 1.

Intraoperative hemodynamics and oxygenation

Mean heart rate (HR) values were comparable between the two groups at baseline and at all intraoperative time points (Table 1). No statistically significant differences were observed at any time point.

**Table 1:** Comparison of Intraoperative Heart Rate (Beats/Minute) Between the Propofol and Sevoflurane Groups Values are expressed as mean ± standard deviation (SD)

Time Point	Propofol (n = 28), Mean ± SD (Beats/Minute)	Sevoflurane (n = 28), Mean ± SD (Beats/Minute)	T-value	P-value
Baseline	78.46 ± 5.64	79.25 ± 5.48	-0.529	0.599
15 minutes	75.93 ± 7.34	76.36 ± 6.04	-0.238	0.812
30 minutes	77.71 ± 5.37	77.39 ± 6.29	0.206	0.838
45 minutes	79.04 ± 7.55	78.93 ± 5.80	0.060	0.953
60 minutes	78.96 ± 6.53	79.43 ± 6.95	-0.258	0.798
75 minutes	81.04 ± 5.57	81.96 ± 6.23	-0.588	0.559
90 minutes	81.75 ± 5.48	82.18 ± 6.63	-0.264	0.793
105 minutes	81.00 ± 6.74	82.18 ± 5.79	-0.702	0.486
120 minutes	82.61 ± 6.34	84.25 ± 6.17	-0.982	0.330

Mean arterial pressure (MAP) values were comparable between groups at all measured time points (Table 2), with no statistically significant differences observed.

**Table 2:** Comparison of Mean Arterial Pressure (MAP, mmHg) Between the Propofol and Sevoflurane Groups Values are expressed as mean ± standard deviation (SD)

Time Point	Sevoflurane (n = 28), Mean ± SD (mmHg)	Propofol (n = 28), Mean ± SD (mmHg)	T-value	P-value
Baseline	88.04 ± 4.57	88.11 ± 4.45	1.129	0.264
15 minutes	87.93 ± 3.89	87.46 ± 4.34	1.107	0.273
30 minutes	89.11 ± 4.99	90.25 ± 4.58	1.198	0.236
45 minutes	88.04 ± 4.99	87.18 ± 4.99	1.616	0.112
60 minutes	87.54 ± 5.95	87.11 ± 5.01	0.688	0.494
75 minutes	88.07 ± 4.80	89.14 ± 4.04	1.498	0.140
90 minutes	88.00 ± 5.06	87.86 ± 4.87	0.528	0.600
105 minutes	89.93 ± 4.70	89.68 ± 2.93	1.361	0.179
120 minutes	88.00 ± 4.29	88.18 ± 4.24	0.733	0.467

Liver function tests (LFTs)

Serum aspartate aminotransferase (AST) and alanine aminotransferase (ALT) levels were measured preoperatively, immediately after extubation and at 24 hours postoperatively (Table 3).

**Table 3:** Comparison of Liver Function Tests (AST and ALT, IU/L) Between the Propofol and Sevoflurane Groups Values are expressed as mean ± standard deviation (SD) AST, aspartate aminotransferase; ALT, alanine aminotransferase

Parameter	Time Point	Propofol (n = 28), Mean ± SD (IU/L)	Sevoflurane (n = 28), Mean ± SD (IU/L)	P-value
AST	Preoperative	27.75 ± 4.96	27.71 ± 4.65	0.978
	Post-extubation	31.18 ± 6.00	32.21 ± 6.17	0.527
	24 hours	34.50 ± 3.19	45.57 ± 4.89	<0.001
ALT	Preoperative	31.75 ± 5.49	30.89 ± 5.51	0.562
	Post-extubation	36.00 ± 7.18	36.18 ± 7.55	0.928
	24 hours	38.32 ± 4.63	55.32 ± 5.96	<0.001

Preoperative AST values were comparable (27.75 ± 4.96 versus 27.71 ± 4.65 IU/L; t = 0.027; df = 54; p = 0.978). Post-extubation AST values were also similar (31.18 ± 6.00 versus 32.21 ± 6.17 IU/L; t = -0.635; df = 54; p = 0.527). At 24 hours, AST levels were higher in Group S compared to Group P (45.57 ± 4.89 versus 34.50 ± 3.19 IU/L; t = -10.037; df = 54; p < 0.001).

Preoperative ALT values were comparable (31.75 ± 5.49 versus 30.89 ± 5.51 IU/L; t = 0.583; df = 54; p = 0.562). Post-extubation ALT values were also similar (36.00 ± 7.18 versus 36.18 ± 7.55 IU/L; t = -0.091; df = 54; p = 0.928). At 24 hours, ALT levels were higher in Group S (55.32 ± 5.96 versus 38.32 ± 4.63 IU/L; t = -11.918; df = 54; p < 0.001).

Reference laboratory ranges were AST of 5-40 IU/L and ALT of 5-40 IU/L.

Renal function tests (RFTs)

Serum creatinine levels were measured at the same time points. Preoperative creatinine values were comparable (0.88 ± 0.07 versus 0.90 ± 0.07 mg/dL; t = -0.956; df = 54; p = 0.344). Post-extubation values showed no significant difference (0.99 ± 0.08 versus 1.00 ± 0.07 mg/dL; t = -0.476; df = 54; p = 0.636).

At 24 hours, creatinine levels were higher in Group S (1.10 ± 0.07 versus 0.89 ± 0.05 mg/dL; t = -12.673; df = 54; p < 0.001). Reference laboratory range for serum creatinine was 0.6-1.2 mg/dL. Table 4 indicates significantly higher serum creatinine in the sevoflurane group at 24 hours, with similar baseline and post-extubation values.

**Table 4:** Comparison of Renal Function (Serum Creatinine, mg/dL) Between the Propofol and Sevoflurane Groups Values are expressed as mean ± standard deviation (SD)

Parameter	Time Point	Propofol (n = 28), Mean ± SD (mg/dL)	Sevoflurane (n = 28), Mean ± SD (mg/dL)	P-value
Serum creatinine	Preoperative	0.88 ± 0.07	0.90 ± 0.07	0.344
	Post-extubation	0.99 ± 0.08	1.00 ± 0.07	0.636
	24 hours	0.89 ± 0.05	1.10 ± 0.07	<0.001

Patient-wise change-from-baseline analysis

Patient-wise changes from preoperative baseline to post-extubation and 24-hour postoperative values were calculated for AST, ALT and serum creatinine. No significant between-group differences were observed in the magnitude of change from baseline to post-extubation for AST, ALT or serum creatinine. However, at 24 hours postoperatively, Group S showed significantly greater increases from baseline in AST, ALT and serum creatinine compared to Group P. These findings indicate that the between-group differences observed at 24 hours were also present when analysed as patient-level changes from baseline, rather than only as pooled absolute postoperative values. Table 5 shows the patient-wise changes from preoperative baseline to post-extubation and 24-hour postoperative values for AST, ALT and serum creatinine, comparing the magnitude of biochemical changes between the propofol and sevoflurane groups.

**Table 5:** Patient-Wise Change-From-Baseline Analysis of AST, ALT and Serum Creatinine Values are expressed as mean ± standard deviation (SD). Changes were calculated for each patient from preoperative baseline to the specified postoperative time point. Between-group comparisons were performed using Welch’s t-test AST, aspartate aminotransferase; ALT, alanine aminotransferase

Parameter	Time Point Change	Group P, Mean ± SD	Group S, Mean ± SD	P-value
AST (U/L)	Preoperative to post-extubation	+3.43 ± 8.49	+4.50 ± 6.67	0.602
AST (U/L)	Preoperative to 24 hours postoperative	+6.75 ± 5.28	+17.86 ± 6.29	<0.001
ALT (U/L)	Preoperative to post-extubation	+4.25 ± 8.62	+5.29 ± 8.69	0.656
ALT (U/L)	Preoperative to 24 hours postoperative	+6.57 ± 6.96	+24.43 ± 8.60	<0.001
Serum creatinine (mg/dL)	Preoperative to post-extubation	+0.10 ± 0.11	+0.09 ± 0.10	0.759
Serum creatinine (mg/dL)	Preoperative to 24 hours postoperative	+0.001 ± 0.08	+0.19 ± 0.08	<0.001

Postoperative recovery and complications

The requirement for rescue antiemetic was also significantly different (χ² = 4.667, df = 1 and p = 0.031). Time to first rescue antiemetic did not differ significantly (χ² = 26.000, df = 18 and p = 0.100).

Sedation scores assessed using the Ramsay Sedation Scale were comparable between groups (χ² = 2.786, df = 2 and p = 0.248) (Table 6).

**Table 6:** Comparison of Ramsay Sedation Scale Scores Between the Propofol and Sevoflurane Groups Values are expressed as number (percentage)

Parameter	Score	Propofol (n = 28), n (%)	Sevoflurane (n = 28), n (%)	P-value
Ramsay Sedation Scale	2	7 (25.0%)	12 (42.9%)	0.248
	3	10 (35.7%)	10 (35.7%)	
	4	11 (39.3%)	6 (21.4%)	

The incidence of postoperative nausea and vomiting (PONV) differed between groups. In Group P, 33.3% of patients experienced PONV compared to 66.7% in Group S (χ² = 4.667, df = 1 and p = 0.029) (Table 7).

**Table 7:** Incidence of Postoperative Nausea and Vomiting (PONV) in the Propofol and Sevoflurane Groups Values are expressed as number (percentage)

PONV Outcome	Propofol (n = 28), n (%)	Sevoflurane (n = 28), n (%)	Total (n = 56), n (%)	P-value
No	20 (71.4%)	12 (42.9%)	32 (57.1%)	0.029
Yes	8 (28.6%)	16 (57.1%)	24 (42.9%)	

## Discussion

The present study found comparable intraoperative hemodynamic stability with propofol- and sevoflurane-based anaesthesia in patients undergoing elective thoracolumbar spine surgery. Heart rate values did not differ significantly between groups at baseline or at any intraoperative time point up to 120 minutes, including the 120-minute value of 82.61 ± 6.34 beats/minute in the propofol group and 84.25 ± 6.17 beats/minute in the sevoflurane group (p = 0.330). Mean arterial pressure also remained comparable, including at 120 minutes, with values of 88.18 ± 4.24 mmHg in the propofol group and 88.00 ± 4.29 mmHg in the sevoflurane group (p = 0.467). Hemodynamic comparisons were limited to the first 120 intraoperative minutes because this represented the common observation period across all included procedures; measurements obtained beyond this duration in longer surgeries were not incorporated into the comparative statistical analysis. These observations are consistent with previous reports demonstrating comparable intraoperative heart rate trends and mean arterial pressure stability between propofol- and sevoflurane-based anaesthesia when titrated according to hemodynamic responses during major surgical procedures [8,10].

Liver enzyme changes differed between groups at 24 hours postoperatively. Baseline AST values were comparable between the propofol and sevoflurane groups (27.75 ± 4.96 versus 27.71 ± 4.65 IU/L; p = 0.978), and post-extubation AST values were also not significantly different (31.18 ± 6.00 versus 32.21 ± 6.17 IU/L; p = 0.527). At 24 hours, AST was significantly higher in the sevoflurane group than in the propofol group (45.57 ± 4.89 versus 34.50 ± 3.19 IU/L; p < 0.001). A similar pattern was observed for ALT. Baseline ALT values were comparable (31.75 ± 5.49 versus 30.89 ± 5.51 IU/L; p = 0.562), as were post-extubation values (36.00 ± 7.18 versus 36.18 ± 7.55 IU/L; p = 0.928), whereas 24-hour ALT was higher in the sevoflurane group (55.32 ± 5.96 versus 38.32 ± 4.63 IU/L; p < 0.001). These results show statistically significant differences in early postoperative transaminase levels. Clinically, interpretation should remain cautious because the observed increases were limited to the first 24 hours, and the study did not evaluate longer-term hepatic outcomes or additional markers such as bilirubin and alkaline phosphatase.

Renal function showed a comparable baseline and immediate postoperative profile, followed by a statistically significant between-group difference at 24 hours. Serum creatinine was similar preoperatively in the propofol and sevoflurane groups (0.88 ± 0.07 versus 0.90 ± 0.07 mg/dL; p = 0.344) and after extubation (0.99 ± 0.08 versus 1.00 ± 0.07 mg/dL; p = 0.636). At 24 hours, creatinine was higher in the sevoflurane group than in the propofol group (1.10 ± 0.07 versus 0.89 ± 0.05 mg/dL; p < 0.001). Although this difference was statistically significant, both group means remained within the stated laboratory reference range of 0.6-1.2 mg/dL. Therefore, the finding is best interpreted as an early biochemical difference rather than evidence of clinically established renal injury.

Postoperative recovery outcomes showed a lower incidence of PONV in the propofol group. PONV occurred in eight of 28 patients receiving propofol and 16 of 28 patients receiving sevoflurane (28.6% versus 57.1%; p = 0.029). Rescue antiemetic requirement also differed significantly between groups (p = 0.031), while time to first rescue antiemetic was not significantly different (p = 0.100). Ramsay Sedation Scale scores were comparable between groups (p = 0.248), suggesting no statistically significant difference in the early postoperative sedation profile. These findings suggest that propofol and sevoflurane provided similar intraoperative hemodynamic stability, while propofol was associated with lower 24-hour AST, ALT and creatinine values and less PONV. The statistical significance of these biochemical differences should be distinguished from clinical significance, as the follow-up period was short, the sample size was limited and most measured values remained within or near laboratory reference ranges. The results support further investigation rather than definitive claims of organ protection. The additional patient-wise change-from-baseline analysis strengthens the interpretation of these findings. Immediate post-extubation changes in AST, ALT and serum creatinine were not significantly different between groups, indicating that early biochemical shifts immediately after anaesthesia were comparable. At 24 hours, however, the magnitude of increase from baseline was significantly greater in the sevoflurane group for AST, ALT and serum creatinine. This confirms that the 24-hour between-group differences were not merely a reflection of pooled postoperative values but were also evident when each patient’s postoperative value was compared to their own preoperative baseline.

The strengths of this study include its prospective randomised comparative design, clearly defined ASA I-II adult study population, standardised perioperative protocol, complete 24-hour follow-up and assessor-blinded postoperative outcome assessment. These design features support internal validity and reduce selection and assessment bias.

Limitations and future directions

This study has several limitations. The sample size was relatively small (n = 56), and follow-up was limited to 24 hours, restricting the assessment of delayed or persistent hepatic and renal changes. Although postoperative assessors and patients were blinded, the anaesthesiologist could not be blinded, which may have introduced performance bias. BIS or equivalent processed electroencephalographic monitoring was not used; the depth of anaesthesia was assessed clinically using hemodynamic parameters. Although anaesthetic agents were administered within predefined protocol ranges, patient-level anaesthetic exposure variables, including cumulative propofol dose, mean and time-weighted propofol infusion rates, fractional inspired and end-tidal sevoflurane concentrations, cumulative volatile exposure and time-weighted MAC exposure, were not prospectively recorded. Therefore, formal dose-response, infusion-rate and concentration-response analyses could not be performed. Accordingly, the observed differences should be interpreted as comparisons between anaesthetic techniques rather than as evidence of exposure-dependent effects. Detailed perioperative variables, including the duration of surgery, intraoperative blood loss, urine output trends, total fluid administration, transfusion requirement and procedure-specific surgical variability, were not comprehensively analysed. Organ function assessment was limited to AST, ALT and serum creatinine, without additional markers such as bilirubin, alkaline phosphatase, estimated glomerular filtration rate or detailed urine output trends. Although statistically significant biochemical differences were observed, most values remained within or near standard laboratory reference ranges and should not be interpreted as definitive evidence of clinically established organ injury.

Future studies should include larger cohorts, longer postoperative follow-up, BIS or equivalent depth-of-anaesthesia monitoring, detailed patient-level anaesthetic exposure recording, comprehensive perioperative variable analysis and broader hepatic and renal biomarkers to clarify whether postoperative biochemical changes are related to anaesthetic class, dose, concentration or perioperative physiology.

## Conclusions

This prospective randomised study shows that both propofol and sevoflurane provide effective and hemodynamically stable anaesthesia for elective thoracolumbar spine surgery. Propofol-based anaesthesia was associated with lower postoperative AST, ALT and serum creatinine levels at 24 hours, along with a lower incidence of postoperative nausea and vomiting (PONV). These findings suggest that propofol may be associated with a more favourable early postoperative biochemical and PONV profile in this clinical setting; however, the clinical significance of these short-term differences remains uncertain. Intraoperative hemodynamic parameters were comparable between the two groups. The results should be interpreted in the context of certain limitations, including the relatively small sample size, short follow-up duration and the inability to fully blind the anaesthesiologist, which may introduce potential bias. Further studies with larger sample sizes, longer follow-up periods and the inclusion of additional biomarkers of organ function are required to validate and extend these findings.
